# A Comparative Study of Two Different Uncinectomy Techniques: Swing-Door and Classical

**Published:** 2012

**Authors:** Ankit A Singhania, Chetan Bansal, Nirali Chauhan, Saurav Soni

**Affiliations:** 1 *Department of otorhinolaryngology, SBKS Medical College, Sumandeep University, Waghodia, Vadodara, Gujarat, India.*

**Keywords:** Classical, Endoscopic surgery, Functional, Sinus, Swing-door technique, Uncinectomy

## Abstract

**Introduction:**

The aim of this study was to determine which technique of uncinectomy, classical or swing door technique.

**Materials and Methods:**

Four hundred eighty Cases of sinusitis were selected and operated for Functional Endoscopic Sinus Surgery (FESS). Out of these, in 240 uncinectomies classical uncinectomy was done whereas in another 240 uncinectomies swing door technique was used. Initially patients were medically managed treated according to their symptoms and prior management. Patients who had received previous adequate medical management were evaluated with CT scan of the sinuses. If disease still persists than they were operated for FESS.

**Results:**

The authors' experience indicates that Functional endoscopic sinus surgery can be performed under local or general anesthesia, as permitted or tolerated. In this review classical technique was used in 240 uncinectomies. Out of this, ethmoidal complex injury was noted in 4 cases, missed maxillary ostium syndrome (incomplete removal) was reported in 12 patients and orbital fat exposure was encountered in 5 patients. As compared to 240 uncinectomies done with swing door technique, incomplete removal was evident in 2 cases and lacrimal duct injury was reported in 3 cases. 'Evidence that underscores how this 'swing door technique' successfully combines 'the conservation goals of the anterior-to-posterior approach and anatomic virtues of the posterior-to-anterior approach to ethmoidectomy of the total 480 uncinectomies operated. Out of which 240 uncinectomies have been performed using the 'swing-door' technique. The 240 uncinectomies performed using classical technique were used as controls. The incidence of orbital penetration, incomplete removal, ethmoidal complex injury and ostium non-identification was significantly less with the new technique. Three lacrimal injuries occurred with the 'swing-door' technique compared to no injuries with classical technique.

**Conclusion:**

The authors recommend swing door technique as it is easy to learn, allows complete removal of the uncinate flush with the lateral nasal wall and allows easy identification of the natural ostium of the maxillary sinus without injuring the ethmoidal complex

## Introduction

Chronic sinusitis (1, 2) is a common problem encountered by otolaryngologists worldwide. Treatment of chronic sinusitis is initially medical and those refractory to medical treatment are treated surgically (3). In 1901 Hirschmann first used a modified Nitzecystoscope to examine the sinuses. Speilberg was then the first to use an endoscope to examine the maxillary sinus through the inferior meatus. However, Maltz coined the term sinoscopy and used an endoscope specially made by Wolfe. The development of compact, straight and angled telescopes, plus the pioneering work of Messerklinger, Wigand and others then sparked an interest in endoscopic sinus surgery (referred to 4-9) and functional endoscopic sinus surgery (FESS) continues to gain popularity among otolaryngologists. Numerous courses have been offered and several papers and books have been written about office evaluation, surgical technique and immediate complications. However as experience is gained, it becomes important to look at long-term results and late complications.

Uncinectomy is the first step performed in FESS (4-6). 

The technique of performing uncinectomy by various methods depends on training and personal preferences. Most surgeons are comfortable with the various techniques of uncinectomy. In this study 480 uncinectomies were performed using the classical uncinectomy technique described by Stammberger or the swing-door technique described by Wormald. The postoperative complications including any comments were recorded. The aim of this study was to determine which uncinectomy technique, Stammberger’s classical technique or the swing-door technique, was preferred by surgeons. 

This paper compares the results of the classical and swing door technique of uncinectomy specifically focusing on their results and conclusions. 

## Materials and Methods

A total of 480 uncinectomies that were performed in patients who underwent FESS were included in the study, of those 240 were uncinectomies using the swing-door technique and 240 were uncinectomies using the conventional technique as a control group. Initially patients were medically managed according to their symptoms and prior course of management. Patients who had received previous adequate medical management were evaluated with a CT scan (10) of the sinuses. If disease still persisted then patients underwent FESS. Only patients with non-polypoid sinusitis were included in the study, those not responding to medical management were also included. The patients were divided into two groups by the use of alternating surgical techniques; the first patient was operated on using the classical technique then the second one was operated on using the swing-door technique and so on.

**Surgical procedure**

Patients underwent FESS under intravenous sedation and local anesthesia and under general anesthesia. The procedure began with decongestion of the nose followed by infiltration of the tissues with a solution of lignocaine 1% with adrenaline. The lignocaine/adrenaline solution was injected into the lateral nasal wall near the uncinate process using a 2 mL syringe with a slightly bent 26-gauge needle to facilitate the injection. Next, the superior inlet and the anterior face of the middle turbinate were injected submucosally with the lignocaine/adrenaline solution. Septoplasty was performed as required. 

**Classical technique**

Uncinectomy was performed via an incision with either the sharp end of a Frere’s elevator or a sickle knife. The incision was placed at the most anterior portion of the uncinate process, which is softer on palpation in comparison to the firmer lacrimal bone, where the nasolacrimal duct is located. Then, Blakesley forceps were used to grasp the free uncinate edge and remove it. Complete uncinectomy is important for subsequent visualization. 

**Swing door technique**

Reverse cutting forceps or backbiting forceps were used in this technique. The inferior free margin overlying the maxillary ostium was cut first and then an incision was made in the superior margin to form a flap from the uncinate which is hinged on the anterior margin and can be moved with an elevator or ball probe. Then, angled trucut forceps were used to grasp the free edge of the uncinate and remove it. This was followed by submucosal removal of the horizontal process of the uncinate and subsequent trimming of the mucosa to fully visualize the maxillary ostium.

Once the uncinate process was removed, the true natural ostium of the maxillary sinus could be identified. The protected eye was palpated at this juncture as necessary to ensure no dehiscence of the lamina papyracea and to confirm the location of the lamina. The natural ostium is typically at the level of the inferior edge of the middle turbinate about one third of the way back. Care should always be taken to avoid penetrating the lamina papyracea.

## Results

In this review the classical technique was used in 240 uncinectomies, which formed the control group. Out of these cases, injury to the ethmoidal complex was noted in 4 patients, missed maxillary ostium syndrome (incomplete removal) was reported in 12 patients and orbital fat exposure was encountered in 5 patients. 240 uncinectomies performed with the swing-door technique, incomplete removal was evident in 2 patients and lacrimal duct injury was reported in 3 patients. The incidence of orbital penetration, incomplete removal, ethmoidal complex injury and ostium non-identification was significantly less with the new technique. However, 3 lacrimal injuries occurred with the swing-door technique compared to no lacrimal injuries with the classical technique. Statistical analysis of the differences in the results of the two techniques showed significant difference. Hence the hypothesis is rejected and proving that the swing-door technique of uncinectomy is better. This evidence underscores how the swing door technique successfully combines the conservation goals of the anterograde (anterior-to-posterior) approach and the anatomic virtues of the retrograde (posterior-to-anterior) approach to ethmoidectomy in the overall group of 480 uncinectomies that were performed.

## Discussion

In our (out patient department) we often encounter patients with recurrent sinusitis requiring revision surgery (5). On endoscopy often some remnant of the uncinate can be seen in many cases. Thus, out of curiosity about the two methods and in an attempt to find a better way of doing an uncinectomy this study was carried out.

The uncinate process can be identified in both coronal and axial CT scans according to its length, inclination (medial or lateral) and its relation to the anterior end of the middle turbinate. The superior attachment of the uncinate determines the pattern of frontal sinus drainage with drainage going into the ethmoid infundibulum when the uncinate process is attached to the fovea ethmoidalis or the middle turbinate or directly into the middle meatus when the uncinate process is attached laterally to the lamina papyracea or the ethmoid cell. Therefore, the antrosuperior attachment of the uncinate process is a good landmark for the frontal sinus ostium. During surgery, the uncinate process can be identified with gentle pressure that reveals its resilience or palpated with a curved probe.

The sickle knife is the traditional instrument for uncinectomy. However, it has some disadvantages such as the occurrence of frequent injury to the inferior turbinate with the proximal part of its cutting edge and injury to the ethmoid bulla with its tip. The main advantage of a sickle knife is that it is a thru-cutting instrument. In contrast, reverse cutting (back biting) forceps are an excellent instrument for uncinectomy. The forceps, particularly when used for the swing-door technique, have many advantages including precise or selective thru cutting of the free edge of the uncinate process with no tendency to injure the inferior turbinate or the ethmoid bulla. The risk of injury to the nasolacrimal duct is not realistic as the newly developed forceps are too delicate to injure the thick bone of the nasolacrimal duct that can be observed on coronal and axial CT scans. Moreover, with precise technique the tip of the cutting blade can easily be seen while cutting. 

The key surgical principles of uncinectomy are as follows: 1) A complete uncinectomy is necessary in order to perform an anterior ethmoidectomy and prevent recurrence of sinusitis; 2) Identification of the maxillary sinus ostium is necessary to find the plane of the lamina papyracea; and 3) It is necessary of perform anterograde (anterior-to-posterior) dissection of the anterior ethmoid cells up to the basal lamella and retrograde (posterior-to-anterior) dissection of the posterior ethmoid cells with no damage to the ethmoidal complex. 

In the traditional anterior-to-posterior uncinectomy described by Messerklinger, an anterior inferior uncinate remnant may remain. This remnant can hide the natural ostium of the maxillary sinus and cause it to be missed. This series of events is what Parsons and colleagues (11) called the “missed ostium sequence”. When performing revision endoscopic sinus surgery, the surgeon might find that the previous middle meatal antrostomy has been placed at the wrong location. 

The traditional method of performing an uncinectomy has a risk of penetration of the lamina papyracea with orbital fat exposure. If the orbital penetration is not recognized, major complications may follow. Sometimes there is a disruption in the ethmoidal complex while removing the uncinate completely. When patients complain of recurrent sinusitis following endoscopic sinus surgery, a recirculation phenomenon in the maxillary sinus may be the cause. Similarly, when the missed maxillary sinus ostium syndrome is identified, endoscopic surgery to connect the natural maxillary ostium with the surgically created middle meatal window can remedy the condition.

## Conclusions

Uncinectomy is an important step in endoscopic sinus surgery. The traditional method of uncinectomy has a risk of injury to the lamina paparycea, also there are more possibilities of incomplete FESS, recurrence and missed maxillary ostium syndrome. Overall, the authors recommend the use of the swing door technique as it is easy to learn, allows complete removal of the uncinate flush with the lateral nasal wall, and allows easy identification of the natural ostium of the maxillary sinus without injuring the ethmoidal complex. The retrograde uncinectomy approach has also been reported to be safe and efficient for identifying the natural ostium of the maxillary sinus during a middle meatal antrostomy. With the development and use of newer forceps and instruments, risk of injury to nasolacrimal duct is also almost negligible.
